# Whole-Genome Sequence of Multidrug-Resistant Bibersteinia trehalosi Strain OADDL-BT1

**DOI:** 10.1128/MRA.01690-18

**Published:** 2019-02-07

**Authors:** Sai Narayanan, Haley Bates, Anthony Confer, Brian Couger, Akhilesh Ramachandran

**Affiliations:** a Oklahoma Animal Disease Diagnostic Laboratory, OSU, Stillwater, Oklahoma, USA; b Department of Veterinary Pathobiology, Center for Veterinary Health Sciences, OSU, Stillwater, Oklahoma, USA; c High Performance Computing Center, OSU, Stillwater, Oklahoma, USA; Georgia Institute of Technology

## Abstract

The genome of a multidrug-resistant strain of Bibersteinia trehalosi isolated from a calf with chronic pneumonia is presented. The draft genome sequences have been deposited at DDBJ/ENA/GenBank.

## ANNOUNCEMENT

Microbial infections resulting in respiratory disease lead to significant economic losses for the North American cattle industry, which are estimated to be in the billions of dollars (1). Bovine respiratory disease is a complex disease involving multiple etiologies, including viral, bacterial, and environmental stress factors.

Bibersteinia trehalosi is a Gram-negative bacterial pathogen known to cause respiratory infections in sheep, goats, cattle, and bison and septicemia in lambs (2, 3). B. trehalosi is a member of the *Pasteurellaceae* family and was previously classified as Pasteurella haemolytica biovar T and then Pasteurella trehalosi (4). Several virulence factors have been identified in B. trehalosi, including fimbriae, polysaccharide capsule, and lipopolysaccharide (5), as well as a leukotoxin, which is considered to be a major virulence factor (6, 7). In addition to virulence factors, increasing antimicrobial resistance has been reported in bacterial respiratory pathogens (8). The presence of antibiotic resistance cassettes and mobile elements that impart resistance to multiple antimicrobial agents has been revealed by whole-genome sequencing of several bovine respiratory pathogens, such as Mannheimia haemolytica and B. trehalosi (2, 9). B. trehalosi infection in cattle is less common compared to that from M. haemolytica or P. multocida. However, an increased frequency of B. trehalosi infection in cattle has been reported (10, 11) and also observed in our laboratory (unpublished).

Here, we present the whole-genome sequence of a B. trehalosi (OADDL-BT1) isolated at the Oklahoma Animal Disease Diagnostic Laboratory from a lung specimen of an 8-month-old Angus cross female calf that died of pneumonia. Bacterial culture was performed on blood agar media (Hardy Diagnostics LLC, Irving, TX) incubated at 37°C in a 5% CO_2_ environment. Bacterial colonies were identified with matrix-assisted laser desorption ionization-time of flight mass spectrometry (MALDI-TOF MS Biotyper, Bruker Daltonics, Billerica, MA). Multiple bacterial pathogens (B. trehalosi, Histophilus somni, and Trueperella pyogenes) were detected. DNA for sequencing was extracted from pure colonies with the EZNA bacterial DNA kit (Omega Bio-Tek, Norcross, GA) following the manufacturer’s recommended protocol.

Genome of the strain OADDL-BT1 was sequenced with the Illumina HiSeq platform using 150 × 2 paired-end reads. A total of 7,131,988 paired-end reads were produced. A quality-filtered sequence using the standard Illumina-recommended protocol data was subsampled to ∼250× coverage and assembled with the short-read de Brujin graph assembly program Velvet (12). The Velvet assembly settings used were a k-mer value of 105, filtering of all contigs that were not supported by 7× coverage, and an expected coverage value of 250×. The resulting assembly had an *N*_50_ scaffold size of 212,914 bp, a maximum scaffold size of 646,523 bp, and a total of 2,408,960 bp. Gene models were produced with the Prodigal prokaryotic gene-calling program (13) using the standard software settings. From the resulting assemblies, 2,238 protein-coding gene models were produced. All predicted protein sequences derived from the assembly were functionally annotated with a combination of homology and a conserved domain search using NCBI BLAST+ (14) and HMMER 3.0 (15) against the Pfam database (16) Standard recommended settings were used for the annotation of each program.

Analysis revealed genes conferring resistance to aminoglycosides [*aph(3′)-Ia/IIb*], sulfonamides (*sul2*), tetracyclines (*tetR*), phenicols (*floR*), and macrolide-lincosamide-streptogramin B families (*msrE*, *mphD*, *erm35*, *erm42*). The genome of Bibersteinia trehalosi OADDL-BT1 will allow further study of antibiotic resistance and functional genomics in a pathologically relevant genus of which only a few sequenced genomes are currently available.

### Data availability.

This whole-genome shotgun project has been deposited at DDBJ/ENA/GenBank under the accession number RRUC00000000. The version described in this paper is accession number RRUC01000000. The sequences have been submitted to the Sequence Read Archive under the accession number PRJNA507647.
